# Beyond the Medical Bill: The Hidden Financial, Employment, and Health Impacts of Caregiving

**DOI:** 10.1093/geroni/igaf122.588

**Published:** 2025-12-31

**Authors:** Barbara Mendez Campos, Christina Matz

## Abstract

Unpaid caregivers provide an essential yet often unrecognized economic foundation for the U.S. healthcare and long-term care systems. Valued at over billions of dollars annually, their contributions surpass Medicaid’s total spending on long-term services and supports. Yet, despite their critical role, caregivers frequently face financial and employment barriers that jeopardize their own well-being. Many reduce work hours, leave the workforce, or forgo career advancement—decisions that come with steep costs, including lost wages, reduced retirement savings, and diminished Social Security benefits. These barriers disproportionately impact women, low-income caregivers, and historically underrepresented racial and ethnic groups, further widening economic and health disparities. This symposium examines the financial, employment, and health implications of caregiving across diverse populations. The first presentation explores the relationship between intergenerational financial support and planned retirement timing among middle-aged and older workers. Using experimental methods, the second presentation investigates and uncovers hiring bias toward family caregivers, finding that hiring penalties for caregivers to older adults may be even greater than hiring penalties toward parents The third presentation quantifies the opportunity cost of family caregiving, estimating lost income, retirement savings, and Social Security benefits for those reducing work hours or leaving the workforce. Using AARP’s latest data and the Urban Institute’s simulation model, it highlights financial disparities by age, income, and care intensity, emphasizing the need for targeted policy solutions. The final presentation examines caregiving-related job disruptions and cognitive outcomes, analyzing how workforce disruptions linked to caregiving contribute to cognitive decline, with disparities evident across gender and racial/ethnic groups. This is a collaborative symposium between the Aging Workforce and Family Caregiving Interest Groups.

